# Human Gut Microbiome Across Different Lifestyles: From Hunter-Gatherers to Urban Populations

**DOI:** 10.3389/fmicb.2022.843170

**Published:** 2022-04-26

**Authors:** Santiago Rosas-Plaza, Alejandra Hernández-Terán, Marcelo Navarro-Díaz, Ana E. Escalante, Rosario Morales-Espinosa, René Cerritos

**Affiliations:** ^1^Centro de Investigación en Políticas, Población y Salud, Universidad Nacional Autónoma de México, Mexico City, Mexico; ^2^Posgrado en Ciencias Biológicas, Facultad de Medicina, Universidad Nacional Autónoma de México, Mexico City, Mexico; ^3^Departamento de Investigación en Tabaquismo y EPOC, Instituto Nacional de Enfermedades Respiratorias Ismael Cosìo Villegas, México City, Mexico; ^4^Laboratorio Nacional de Ciencias de la Sostenibilidad (LANCIS), Instituto de Ecología, Universidad Nacional Autónoma de México, Mexico City, Mexico; ^5^Departamento de Microbiología y Parasitología, Facultad de Medicina, Universidad Nacional Autónoma de México, Mexico City, Mexico

**Keywords:** meta-analysis, lifestyles, 16S r RNA, gut microbiome, bacterial diversity

## Abstract

Human lifestyle and its relationship with the human microbiome has been a line of research widely studied. This is because, throughout human history, civilizations have experienced different environments and lifestyles that could have promoted changes in the human microbiome. The comparison between industrialized and non-industrialized human populations in several studies has allowed to observe variation in the microbiome structure due to the population lifestyle. Nevertheless, the lifestyle of human populations is a gradient where several subcategories can be described. Yet, it is not known how these different lifestyles of human populations affect the microbiome structure on a large scale. Therefore, the main goal of this work was the collection and comparison of 16S data from the gut microbiome of populations that have different lifestyles around the world. With the data obtained from 14 studies, it was possible to compare the gut microbiome of 568 individuals that represent populations of hunter-gatherers, agricultural, agropastoral, pastoral, and urban populations. Results showed that industrialized populations present less diversity than those from non-industrialized populations, as has been described before. However, by separating traditional populations into different categories, we were able to observe patterns that cannot be appreciated by encompassing the different traditional lifestyles in a single category. In this sense, we could confirm that different lifestyles exhibit distinct alpha and beta diversity. In particular, the gut microbiome of pastoral and agropastoral populations seems to be more similar to those of urban populations according to beta diversity analysis. Beyond that, beta diversity analyses revealed that bacterial composition reflects the different lifestyles, representing a transition from hunters-gatherers to industrialized populations. Also, we found that certain groups such as *Bacteoidaceae*, *Lanchospiraceae*, and *Rickenellaceae* have been favored in the transition to modern societies, being differentially abundant in urban populations. Thus, we could hypothesize that due to adaptive/ecological processes; multifunctional bacterial groups (e.g., *Bacteroidaceae*) could be replacing some functions lost in the transition to modern lifestyle.

## Introduction

The human microbiome has been proposed as a fundamental key for maintaining health, including the regulation of the immune system, food digestion, synthesis of vitamins, and protection against pathogens (Selber-Hnativ et al., 2017). Specifically for the gut microbiome, it has been documented that alterations or dysbiosis in the microbial communities have consequences on human health, and have been associated with inflammatory bowel disease, diabetes, and arthritis (Ding et al., 2019). Thus, given the directed or undirected consequences that changes in the microbiome have for human health, it is crucial to understand what factors affect the diversity and composition of the human microbiome in order to get insights into the relationship between human health and microbiomes.

In particular, human lifestyle and its relationship with the gut microbiome have been widely studied (De Filippo et al., 2010; Yatsunenko et al., 2012; Schnorr et al., 2014; Clemente et al., 2015; Obregon-Tito et al., 2015; Jha et al., 2018; Hansen et al., 2019). Throughout human history, civilizations have experienced different environments and lifestyles, which could have led to changes in the microbial communities in the human gut (Jha et al., 2018). For example, the transition from hunter-gatherers to industrialized populations (urbanization) represents important modifications in diet, hygiene practices, health care (including antibiotics), access to resources, and the way that humans interact with the environment (Warinner et al., 2015; Jha et al., 2018). The urbanization process has also promoted globalized supply chains with an increase in the consumption of dairy products, processed foods, and high fat (Jennings et al., 2015). Moreover, it is known that urban populations are less exposed to natural habitats than rural ones, modifying the interaction with microorganisms in the environment (Callewaert et al., 2020). Altogether, the factors involved in the different lifestyles of human populations drive important shifts in the composition, diversity, and structure of the gut microbiome, which in turn have consequences in human health.

Previous studies have found that the gut microbiomes associated with industrialized populations are less diverse and have different microbial compositions than those from traditional lifestyles (Clemente et al., 2015). For instance, it has been shown several times that the microbiomes from human populations living in industrialized areas are dominated by the genus *Bacteroides*, while hunter-gatherer populations exhibit a high abundance of the genera *Prevotella* and *Treponema* (Schnorr et al., 2014; Obregon-Tito et al., 2015). It is important to highlight that such genera are commonly associated with particular functions in the gut microbiome. Specifically, Bacteroides has been associated with high-fat consumption (Singh et al., 2020), whereas *Prevotella* and *Treponema* have been linked to high-fiber consumption (De Filippo et al., 2010; Ayeni et al., 2018). Metagenomic analysis also has revealed that the microbial communities associated with industrialized populations present more antibiotic resistance genes than traditional populations (Obregon-Tito et al., 2015; Wibowo et al., 2021). More recent investigations also have found that traditional lifestyles have a higher abundance of mobile genetic elements, probably as adaptations to the seasonal variations (Wibowo et al., 2021).

Although the changes in abundance of microbial groups associated with particular functions seem to be consistent in the studies that compare industrialized and non-industrialized populations (Mancabelli et al., 2017) much remains to be understood between the relationship of the human gut microbiome and human lifestyles. That is, most large-scale studies (meta-analysis) have tried to show the differences between industrialized and non-industrialized populations without taking into account that, within traditional lifestyles such as hunter-gatherer, pastoralist, and agricultural populations, different human practices could be reflected in the composition of the gut microbiome. That is to say, we still don’t know if there are differences in the gut microbiome of populations that carry out activities of domestication of plants, animals, or in the populations that depend on the resources that are available in the environment without an established production system.

Despite the importance of testing how the urbanization process and different lifestyles can impact the composition of the human gut microbiome, no systematic analysis of the reported data exists to date that take into account the different traditional lifestyles as distinct categories (hunter-gatherers, pastoral, agropastoral, agricultural, and urban populations). Thus, we propose a meta-analysis approach that allows us to integrate a large amount of data and identify patterns among different studies that are constructed under the same theoretical framework. The main goal of this meta-analysis was the comparison of the gut microbiome of populations that have different lifestyles across different geographical regions. For this meta-analysis, we analyzed publicly available sequences of 16S rRNA gene from different studies comparing the gut microbiome of human populations. The samples include meta-amplicon sequences of gut microbiomes of hunter-gatherers, pastoral, agropastoral, agricultural, and urban populations.

The collected data allowed us to test if the urbanization process and lifestyle can impact the composition of the gut microbiome. We calculated alpha and beta diversity indices and compared them according to the population’s lifestyle. Also, we identified deferentially abundant microbes between the microbial gut microbiome of human populations with different lifestyles that could be biomarkers. Based on the Phylogenetic Diversity Index we found that, in general, industrialized populations present less diversity than those from non-industrialized populations. Moreover, the PCoA with unweighted UniFrac distance and Beta Dispersion analyses confirm that different lifestyles exhibit distinct beta diversity, representing the transition from hunter-gatherers to urban populations.

## Materials and Methods

### Data Collection

To compare different lifestyles around the world, raw sequences of 16S rRNA gene of human gut microbiome were downloaded and processed. The analysis included sequences of gut microbiome from hunter-gatherers (Jha et al., 2018; Smits et al., 2018; Hansen et al., 2019; Rubel et al., 2020), pastoral populations (Angelakis et al., 2016; Li H. et al., 2018; Hansen et al., 2019; Rubel et al., 2020), agropastoral populations (Quagliariello et al., 2019; Rubel et al., 2020), traditional agricultural populations (Yatsunenko et al., 2012; Jha et al., 2018; Ruggles et al., 2018) and people who live in urban areas (Yatsunenko et al., 2012; Aly et al., 2016; Angelakis et al., 2016; Bressa et al., 2017; Fujio-Vejar et al., 2017; Girard et al., 2017; McDonald et al., 2018; Quagliariello et al., 2019). Information about geographical region, sex, and age of each individual are available at Supplementary Table 1.

### Sample Selection Criteria and Establishment of Lifestyle

To control variables that have been documented as factors that influence the human gut microbiome, exclusion criteria were established for the selection of samples and construction of the database. We took a minimum number of six samples per population with individuals over 18 years old. Individuals reported with metabolic diseases were removed from the analysis. In the case of studies where healthy controls were analyzed against individuals with some type of disease, only healthy individuals were selected. Studies that employed sequencing techniques other than the Illumina platform were also excluded. For this analysis, all 16S gene regions were included (Supplementary Table 1). To categorize human populations lifestyles we used the classification of Food and Agricultural Organization (FAO), and information reported in the individual analyzed studies. We categorized the lifestyles as follows: (a) Hunters-gatherers: people whose main activity is foraging and hunting resources that are around their environments (Galvin and Beeton, 2013), (b) Pastoral: populations whose main activity is raising livestock (Little, 2015; FAO, 2016), (c) Agropastoral: populations that mix small agriculture and livestock (Little, 2015; FAO, 2016), and (d) Agricultural: populations that practice traditional agriculture mainly crop-based and in some cases with small livestock (Edwards et al., 1988).

### Data Processing

16S rRNA raw sequences (.fastq files) were downloaded from public repositories (e.g., NCBI, MG-Rast, Github) and analyzed with the Quantitative Insights Into Microbial Ecology (QIIME2) platform (Bolyen et al., 2019) version 2020.2. Processing was done with single-end sequences and individually for each article dataset. Sequences denoising and quality filtering, including primers and adapters trimming, were performed with the DADA2 plug-in (Callahan et al., 2016). We established a cutoff between 100 and 150 nt for all sequences depending of their quality and primers position. The taxonomic assignment of the Amplicon Sequence Variants (ASVs) was carried out independently for each article dataset with the “sklearn-base” classifier using the latest version of Greengenes 13_8 database (McDonald et al., 2012) and with a 99% identity threshold. Samples with less than 7,000 sequences after quality filtering were excluded from the analyses.

Since we analyzed different variable regions of the 16S rRNA gene, the results of each data set were joined in a single feature table and then processed with the Fragment-Insertion QIIME2 plug-in (Janssen et al., 2018) using the reference database sepp-refs-gg-13-8 with default parameters. This plug-in is based on SATé-Enabled Phylogenetic Placement (SEPP) and has been reported as a solution when analyzing different variable regions of the 16S gene (Janssen et al., 2018). The process resulted in a phylogenetic tree that was used to filter the feature table for the subsequent analyses. All analyses related to sequence data processing were performed in QIIME2 (v.2020-2). Finally, all ASVs that match with mitochondria and chloroplast were removed and the number of sequences was standardized by applying a rarefaction of 7,000 (700 permutations) sequences per sample with R’s phyloseq package (McMurdie and Holmes, 2013).

### Statistical Analyses

#### Alpha Diversity Analysis

To compare the gut microbiome diversity of populations living different lifestyles, we calculated the Phylogenetic diversity (PD) and Shannon-Wiener indexes. The indexes were calculated with the “vegan” and “PhyloMeasures” packages in R (Oksanen et al., 2008; Tsirogiannis and Sandel, 2016). Statistical significant differences in alpha diversity between microbial communities were calculated with the Wilcoxon rank-sum test using “vegan” package in R (Oksanen et al., 2019).

#### Beta Diversity Analysis

For beta diversity, a Principal Coordinate Analysis (PCoA) with unweighted UniFrac was performed at the family level with “phyloseq” R package (McMurdie and Holmes, 2013). Permutation analysis of variance test (PERMANOVA) with unweighted UniFrac matrix (999 permutations) was performed to detect global differences between lifestyles with “vegan” R package. In addition, pairwise differences in beta diversity were analyzed with a multivariate dispersion analysis, using the betadisper function with the unweighted UniFrac distance matrix in “vegan” R package (Oksanen et al., 2019). Then, a pairwise Wilcoxon rank-sum test was employed to detect statistical significant differences in beta dispersion among lifestyles. Moreover, we employed different approaches to detect differentially abundant groups. First, pairwise comparison of differentially abundant groups between lifestyles was carried out with “DESeq2” package (Love et al., 2014), using “poscounts” size factor estimation and default settings. Bacterial groups with a *p-*adjusted < 0.01 were taken for the analysis (Supplementary Figures 3, 4). Secondly, a Linear Discriminant Analysis (LDA) with effect size (LEfSe) was performed at the family level in the MicrobiomeAnalyst platform (Dhariwal et al., 2017). In LEfSe, only features with an LDA score higher than 2 and a *p-*adjusted < 0.01 were used for the analysis. The bacterial groups that were differentially abundant in both analyses (DESeq2 and LEfSe) were represented through an alluvial graphic plotted in R. Stacked bar plots of the 20 most abundant families were performed with “phyloseq” R packages (McMurdie and Holmes, 2013).

Finally, in order to corroborate the patterns found at the family level, all the diversity analyses described above were also carried out with the same parameters at the genus level (Supplementary Figures 2, 5–7).

## Results

### Study Populations

To analyze the gut microbiome of populations living different lifestyles, we search for publicly available sequences of the 16S gene data. According to the exclusion criteria, we collected data from 14 studies with a total of 568 individuals. These data represent three populations of hunters-gatherers (*n* = 187), four pastoral populations (*n* = 67), two agropastoral populations (*n* = 53), six populations of agriculturists (*n* = 99), and 10 populations that live in urban areas (*n* = 162). We included in the study the v1-v2, v3-v4, v4, and v4-v5 16S variable regions (Supplementary Table 1).

### Alpha Diversity Across Different Lifestyles

**FIGURE 1:**
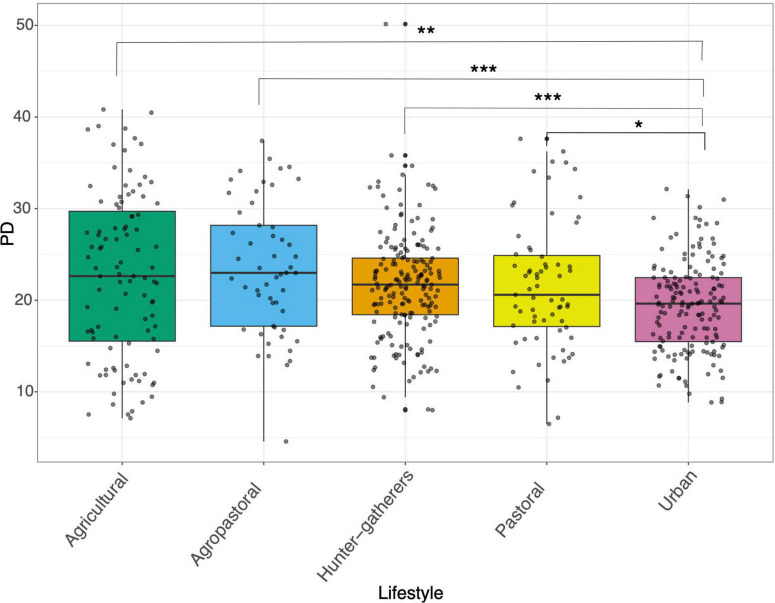
Alpha diversity of human gut microbiome across lifestyles. Boxplot of Phylogenetic diversity analysis (PD) between hunter-gatherers, agricultural, agropastoral, pastoral, and urban populations. Asterisks denote statistical significant differences given by Wilcoxon rank-sum test (*p < 0.05, **p < 0.01, ***p < 0.001, ****p < 0.0001).

Based on the Phylogenetic Diversity index (PD), the gut microbiome of urban populations presented the lowest values of alpha diversity, being significantly less diverse than all other lifestyles (Figure 1). Also, it is important to highlight that within traditional lifestyles there were no significant differences in terms of alpha diversity (Figure 1). In contrast, the Shannon-Winner index showed that hunter-gatherers and agropastoral populations were significantly more diverse than pastoral, agricultural, and urban populations (Supplementary Figure 1).

### Beta Diversity Across Different Lifestyles

**FIGURE 2:**
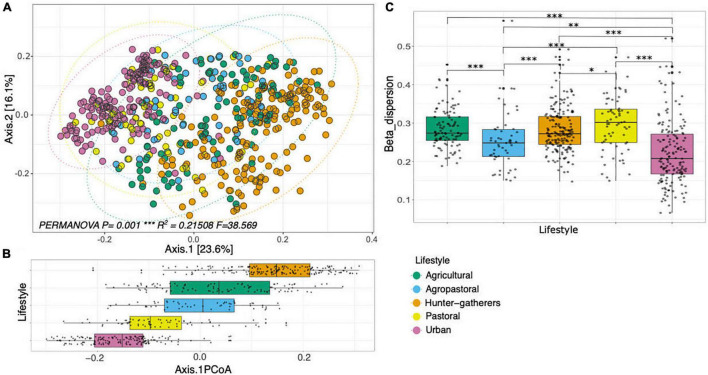
Beta diversity analysis of human gut microbiome between lifestyles. **(A)** Principal Coordinates Analysis (PCoA) with unweighted UniFrac distance at the family level. **(B)** Boxplots of the distribution of each lifestyle across axis 1 of the PCoA. **(C)** Boxplot of the Multivariate Beta Dispersion analysis comparison of different lifestyles. Asterisks denote statistical significant differences given by Wilcoxon rank-sum test (*p < 0.05, ^**^p < 0.01, ^***^p < 0.001, ^****^p < 0.0001).

Regarding beta diversity, the PCoA with unweighted UniFrac distance at the family level showed a the microbiome structure is slightly different according to lifestyles of hosts and explained almost 40% of the total variation (axis1 = 16.1% and axis2 = 23.6%) (Figures 2A,B). These differences were corroborated with the PERMANOVA test (*p* = 0.001, *R*^2^ = 0.21508, *F* = 38.5) and the beta dispersion analysis, which showed that all lifestyles were significantly different (Figure 2C). Also in the PCoA figure, we appreciated a shift of diversity from hunter-gatherers to urban populations. For instance, while hunter-gatherers and urban population are in opposite sites of the of the plot, pastoral, agropastoral, and agricultural populations hosts represent intermediate states of microbiome structure. Finally, it is worth to mention that we found the same patterns of beta diversity at genus level (Supplementary Figure 2).

**FIGURE 3:**
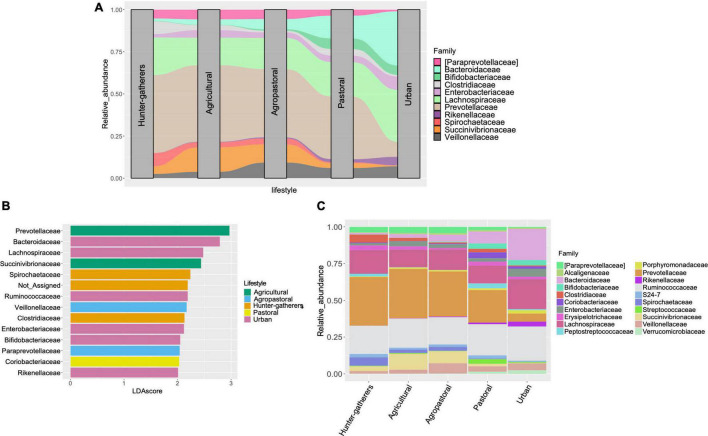
Differentially abundant bacterial families between lifestyles. **(A)** Alluvial plot representing the differentially abundant families among lifestyles according to LEfSe and DEseq2 analyses. **(B)** Linear Discriminant Analysis with Effect Size (LEfSe) at the family level between lifestyles. Only features with LDA score higher than 2.0 and *p* < 0.05 were included. **(C)** Stacked barplot of relative abundance of the top 20 most abundant bacterial families in each lifestyle.

On the other hand, both the DEseq2 and LEfse analyses at the family level (Figure 3C and Supplementary Figures 3, 4) revealed some patterns in urban and traditional lifestyles that are summarized in the alluvial plot (Figure 3A) and that were also recovered at the genus level (Supplementary Figures 5–7). For instance, we found that the bacterial families *Bacteroidaceae, Bifidobacteriaceae, and Rikenellaceae* were differentially abundant in urban and pastoral populations compared to the other traditional lifestyles (Figures 3A–C and Supplementary Figures 3, 4). Contrary, *Prevotellaceae, Paraprevotellaceae, Succinivibrionaceae*, and *Spirochaetaceae*, were more abundant in hunter-gatherers, agricultural, and agropastoral populations (Figure 3A). Besides, in LEfse analysis, we found that hunter-gatherers showed a particularly high abundance of *Spirochaetaceae* while agricultural populations present a high abundance of *Succinivibrionaceae* and *Prevotellacea*. Contrary, for urban populations were *Bacteroidaceae, Lachnospiraceae, Bifidobacteriaceae, Rikenellaceae*, and *Enterobacteriaceae* (Figure 3B).

## Discussion

Different studies have suggested that human lifestyle is involved in the diversity and composition of the human gut microbiome (De Filippo et al., 2010; Yatsunenko et al., 2012; Schnorr et al., 2014; Clemente et al., 2015; Martínez et al., 2015). However, few studies have tested this hypothesis through systematic collection and analysis of information from populations living in different lifestyles. To determine if, traditional lifestyles and urban populations show previously reported diversity patterns (out of the categorization of industrialized and non-industrialized populations), we performed a meta-analysis focused on collecting information and analyzing 16S data of the human gut microbiome.

### From Traditional Lifestyles to Urban Populations

As previously reported, we found that populations with traditional lifestyles exhibit significantly higher alpha diversity than populations living in urban areas or with some degree of industrialization (Figure 1). This finding supports the hypothesis that the urbanization process involves a series of changes related to the modification of the alpha and beta diversity of the human gut microbiome.

Further, by recognizing that non-industrialized lifestyles populations can be separated into sub categories new patterns arise. For instance, we found that some categories such as pastoralist showed less alpha diversity than all other traditional lifestyles (Figure 1). This result has been previously reported in pastoral populations compared to hunter-gatherers and agropastoral populations (Hansen et al., 2019). Historically, pastoralism was practiced in regions where the lands were poor to carry out other activities like agriculture, therefore animal products were considered as the main source of food (e.g., milk, meat, and other animal-derived products) (Little, 2015). It has been shown that a high-fat diet is associated with a decrease in alpha diversity, while a diet rich in fiber is generally associated with increased alpha diversity (Wan et al., 2019). Thus, the limited variety of food resources coupled with a high-fat diet could be influencing the low alpha diversity in pastoral populations, highlighting that a traditional lifestyle does not necessarily lead to a greater diversity of the gut microbiome. Overall, considering the findings described above, it makes sense that populations that are known to have a high-fiber diet like agricultural populations showed the highest values for alpha diversity, whereas populations with higher intakes of animal products like urban and pastoral populations presented the lowest alpha diversity (Figure 1).

On the other hand, by separating the populations into different lifestyles we observed changes in the microbiome composition that could be associated with the transition of lifestyles. Most changes are related with bacterial groups [at family (Figure 3) and genus level (Supplementary Figure 2)] that are known to degrade plant polysaccharides and are associated with a high-fiber diet, such as *Prevotellaceae (g_Prevotella), Paraprevotellaceae*, *Spirochetaceae (g_Treponema), Clostridiaceae*, and *Succinivibrionaceae (g_Succinivibrio)* (De Filippo et al., 2010; Ormerod et al., 2016; Guo et al., 2020; Tett et al., 2021). These polysaccharides-degrading groups were significantly abundant in all traditional lifestyles but less present in pastoral populations and urban populations (Figure 3). In terms of human health, it is important to mention that members of the family *Prevotellaceae (g_Prevotella) and Succinivibrionaceae (g_Succinivibrio)* are able to ferment complex polysaccharides to produce short-chain fatty acids (SCFAs) that play an important role in colon health (Alemao et al., 2020). Contrary, it seems that the microbiomes associated with urban populations have suffered a depletion of these bacterial families but an enrichment of families correlated with high-fat and high-animal protein diets, such as *Bacteroidaceae, Lachnospiraceae*, and *Rikenellaceae* (Okeke et al., 2014; Lecomte et al., 2015; Vacca et al., 2020). It is important to highlight that these groups also have metabolic functions associated with the degradation of complex polysaccharides (Flint and Duncan, 2014; Vacca et al., 2020). However, it has been reported that in the absence of nutrients found in high-fiber food (galacto- and fructo-oligosaccharides, resistant starch), some bacterial groups adapt to obtain energy through digestion of the mucus barrier (Alemao et al., 2020). This is the case of *Bacteroidaceae, Lachnospiraceae*, and *Rikenellaceae* that have been reported as main mucosal-sugar generalists (Pereira et al., 2020). These groups probably have to adapt when urban populations transition from a high-fiber diet to a high-fat diet. Nevertheless, alterations of mucosa membranes and the lack of foods rich in fiber have been associated with immune disorders such as inflammatory bowel disease, obesity, and diabetes (Alemao et al., 2020). Moreover, *Lachnospiraceae and Bacteroidaceae* families have been also related to obesity, colon cancer, and diabetes (Alemao et al., 2020; Vacca et al., 2020), which are considered diseases of western lifestyles (Carrera-Bastos et al., 2011) and nowadays more than a billion people in the world has been affected by metabolic syndrome (Saklayen, 2018).

Opposite to *Prevotellaceae*, the *Bacteroidaceae* group seems to have been favored in the transition to modern lifestyles. While in hunter-gatherers *Bacteroidaceae* is almost absent, in the transition to plant domestication (agricultural populations) the abundance of this group increases (Figure 3A). However, in the transition to animal domestication (pastoral populations), we appreciate a significant increase of such group, culminating in modern societies where *Bacteroidaceae* was the differentially abundant group (Figure 3B and Supplementary Figure 2). An important characteristic of some members of the *Bacteroidaceae (e.g., Bacteroides)* and *Rikenellaceae* family is the capability to tolerate bile, which is linked to higher consumption of animal products (Graf, 2014; Tomova et al., 2019). Additionally, metatranscriptomic analyses have shown that *Bacteroidaceae* is involved in almost all bacterial functional genes categories in the active gut microbiome (Yap et al., 2014), including the degradation of complex carbohydrates (Flint et al., 2012). In this sense, the *Bacteroidaceae* group could have increased their abundance due to the loss of bacterial groups that are not tolerant to high bile content, in addition to its capability to perform different metabolic functions in the human intestine. Moreover, members of the *Bacteroidaceae (g_Bacteroides)* family as well as other genera such as *Akkermansia* [found highly abundant in urban populations in this study (Supplementary Figure 2)] have been adapted to degrade the mucus barrier in the gut. This probably resulted in a drastic increase of *Bacteroidaceae* abundance in the transition to modern lifestyle.

Similarly, animal domestication could lead to the increase of other groups such as the *Bifidobacterium*. In particular, the analysis at the genus level revealed that pastoral and urban populations presented significantly more abundance of this group than all other lifestyles (Supplementary Figure 2). *Bifidobacterium* is dominant in the gut of healthy breast-fed infants but in adulthood their abundance decreases (Arboleya et al., 2016). Nevertheless, the consumption of dairy products in adulthood has promoted the enrichment of such groups, since various species of *Bifidobacterium* utilize lactose as their main source of energy (Li X. et al., 2018). Additionally, has been reported that *Bifidobacterium* can inhibit the growth of *Treponema* species in the gut (Belkacemi et al., 2020) as we found in the differentially abundant analyses at the genus level (Supplementary Figure 2). *Treponema* genus is a member of the family *Spirochaetaceae that* is associated with high-fiber diet (Angelakis et al., 2019). This could explain why the abundance of the family *Spirochaetaceae* is considerably low in urban and pastoral populations, whereas in hunter-gatherers and agricultural populations the abundance of *Spirochaetaceae* is higher than *Bifidobacteriaceae* (Figure 3A). Altogether, these findings could support the antagonistic interaction between *Treponema-Bifidobacteirum.*

The PCoA analysis performed by lifestyle at both family and genus level (Figure 2A and Supplementary Figure 2) showed that the different hosts’ lifestyles are recovered in the microbiome structure. Particularly, the pattern of change in the microbiome structure seems to recover the transition of the human populations, from the passage of hunter-gatherers to the domestication of plants, followed by the domestication of animals and finally the modern urban populations. However, the observed differences in microbiome composition according to lifestyles (Figure 3C) could be mainly driven by changes in diet composition (e.g., high-fat or high-fiber diets). For example, populations that have a higher consumption of animal-derived products, such as urban, pastoral and agropastoral populations were closer in the PCoA. Contrary, the populations that are known to have a high-fiber diet such as agricultural and hunter-gatherers were closer to each other but more distant to urban populations. Furthermore, we can appreciate that between the categories associated with a high-fat diet (pastoral and agropastoral) there are some differences in the intakes of animal products that seem to be reflected in the PCoA. For instance, pastoral populations that have a higher fat diet compared to other traditional lifestyles were closer or even overlapped with urban populations, followed by agropastoral populations. In the case of agropastoral populations, they have mixed practices between agriculture and pastoralism, which results in higher intakes of foods rich in fiber compared to pastoral or urban populations. That could be the reason why agropastoral populations were shown as an intermediate state between pastoral and agricultural populations in the PCoA. In this context, all the results obtained in this meta-analysis seem to corroborate the hypothesis that, over other variables, diet could have a primary role in shaping the gut microbiome (De Filippo et al., 2010).

## Conclusion

Our results support the hypothesis that populations that have undergone a process of urbanization present less diversity in the gut microbiome. According to our meta-analysis based on 16S gene sequences, we could confirm that beyond the industrialized and non-industrialized categories, the different lifestyles established in this study reflect specific structural patterns in the microbial communities of the human gut. Moreover, the major differences between lifestyles were found in the proportion of groups associated with high-fiber and high-fat diets, indicating that diet plays a major role in the composition of human gut microbiome. In particular, the transition from traditional to modern lifestyle seems to impact the gut microbiome by decreasing diversity and promoting the growth of particular microbial groups. That is the case of *Bacteroidaceae* family which represents one of the most abundant groups in urbanized populations and is known to be involved in almost all bacterial functional genes categories. Thus, we could hypothesize that due to adaptive/ecological processes; multifunctional bacterial groups (e.g., *Bacteroidaceae*) could be replacing some functions lost in the transition to modern lifestyle. Finally, it is important to mention that the integration of more detailed and precise information of populations and individual samples in future gut microbiome studies is crucial to perform meta-analyses that can integrate and analyze other variables related to the individuals.

## Data Availability Statement

The original contributions presented in the study are included in the article/Supplementary Material, further inquiries can be directed to the corresponding author/s.

## Author Contributions

SR-P, AE, RM-E, and RC conceived and designed the project. SR-P and AH-T performed the bioinformatics and statistical analyses. SR-P, AE, RM-E, RC, AH-T, and MN-D wrote the manuscript. RC supervised the project and led the team. All authors discussed the results and commented on the manuscript.

## Conflict of Interest

The authors declare that the research was conducted in the absence of any commercial or financial relationships that could be construed as a potential conflict of interest.

## Publisher’s Note

All claims expressed in this article are solely those of the authors and do not necessarily represent those of their affiliated organizations, or those of the publisher, the editors and the reviewers. Any product that may be evaluated in this article, or claim that may be made by its manufacturer, is not guaranteed or endorsed by the publisher.

## Supplementary Material

Supplementary Table 1 Metadata associated to the analyzed samples (sample accession, lifestyle, 16S variable region, geographic information, sex, and age).
