# Corrigendum: Human gut microbiome across different lifestyles: from hunter-gatherers to urban populations

**DOI:** 10.3389/fmicb.2024.1435763

**Published:** 2024-07-03

**Authors:** Santiago Rosas-Plaza, Alejandra Hernández-Terán, Marcelo Navarro-Díaz, Ana E. Escalante, Rosario Morales-Espinosa, René Cerritos

**Affiliations:** ^1^Centro de Investigación en Políticas, Población y Salud, Universidad Nacional Autónoma de México, Mexico City, Mexico; ^2^Posgrado en Ciencias Biológicas, Facultad de Medicina, Universidad Nacional Autónoma de México, Mexico City, Mexico; ^3^Departamento de Investigación en Tabaquismo y EPOC, Instituto Nacional de Enfermedades Respiratorias Ismael Cosìo Villegas, México City, Mexico; ^4^Laboratorio Nacional de Ciencias de la Sostenibilidad (LANCIS), Instituto de Ecología, Universidad Nacional Autónoma de México, Mexico City, Mexico; ^5^Departamento de Microbiología y Parasitología, Facultad de Medicina, Universidad Nacional Autónoma de México, Mexico City, Mexico

**Keywords:** meta-analysis, lifestyles, 16S r RNA, gut microbiome, bacterial diversity

In the published article, there was an error concerning the affiliations for author Santiago Rosas-Plaza. As well as having affiliation one, they should also have *Posgrado en Ciencias Biológicas, Facultad de Medicina, Universidad Nacional Autónoma de México, Mexico City, Mexico*.

In the published article, there was an error in the Funding statement. The Funding statement was displayed as “This manuscript was part of the doctoral project of SR-P, who thanks the Posgrado en Ciencias Biologicas, UNAM and acknowledges the doctoral scholarship supported by the Consejo Nacional de Ciencia y Tecnología (CONACyT) (CVU: 776199).” The correct Funding statement appears below.

