# Supplementary material for: Human Gut Microbiome Across Different Lifestyles: From Hunter-Gatherers to Urban Populations
Source: Front Microbiol. 2022 Apr 26;13:843170. doi: 10.3389/fmicb.2022.843170 (PMC9087276; doi:10.3389/fmicb.2022.843170)
Supplement: Supplementary file 1 [file Data_Sheet_1.docx]

**SUPPLEMENTARY MATERIAL**

A


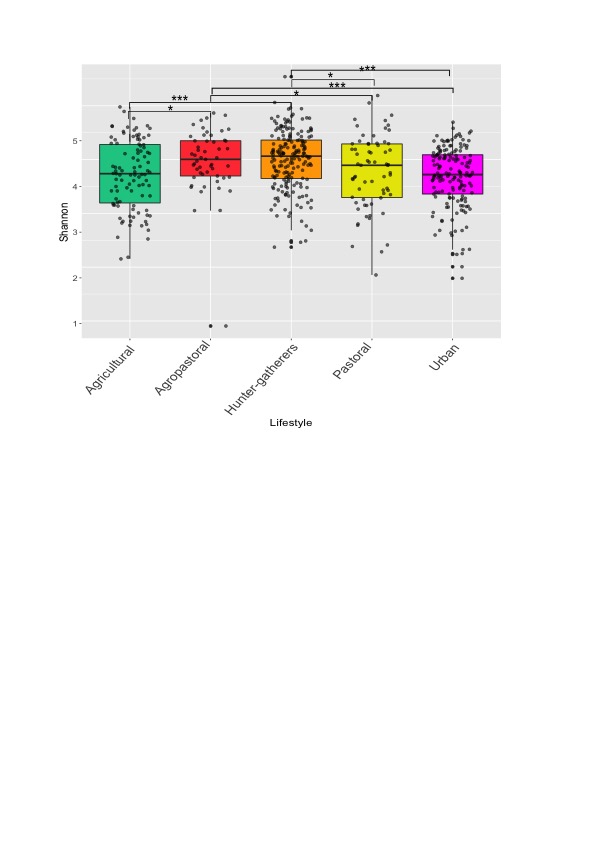


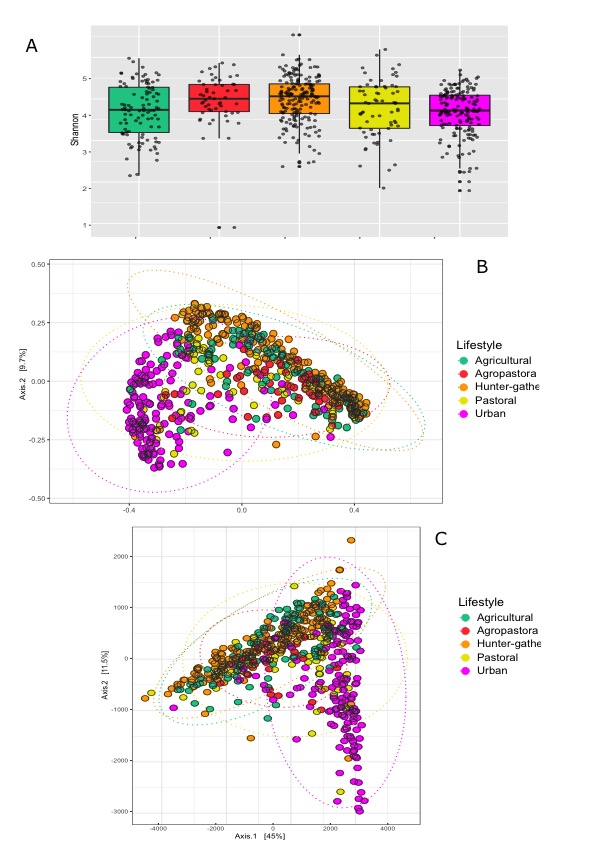


**Supplementary Figure S1. A)** Shannon diversity index across lifestyles (NS: p > 0.05, * p <= 0.05, ** p <= 0.01, *** p <= 0.001, **** p <= 0.0001) B) PCoA with Jaccard distance across lifestyles at family level. C) PCoA with euclidean distance across lifestyles at family level.


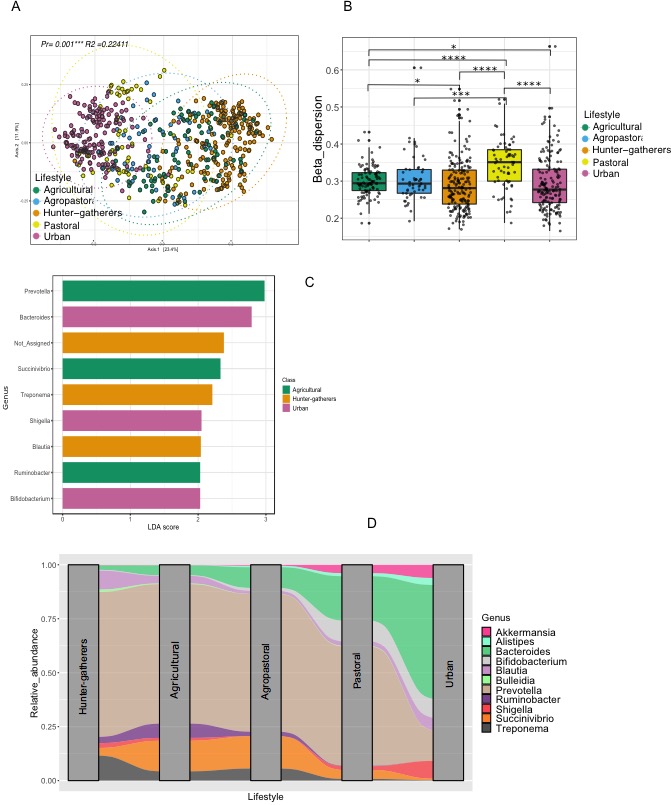
**Supplementary Figure S2. Alpha and beta diversity analyses among lifestyles at the genus level.** A) PCoA with unweighted UniFrac distance with different lifestyles at the genus level. B) Multivariate beta dispersion analysis among lifestyles at the genus level. C) Linear Discriminant Analysis with Effect Size (LEfSe) between lifestyles at the genus level. D) Alluvial plot of the bacterial genus that were differentially abundant according to LEfSe and DESeq2 analysis.

**Supplementary Figure S3. Pairwise comparison of differentially abundant families (DESeq2 analysis) between different lifestyles.**

**Supplementary Figure S4. Pairwise comparison of differentially abundant families (DESeq2 analysis) between different lifestyles.**

**Supplementary Figure S5. Pairwise comparison of differentially abundant groups (DESeq2 analysis) between different lifestyles at the genus level.**

**Supplementary Figure S6. Pairwise comparison of differentially abundant groups (DESeq2 analysis) between different lifestyles at the genus level.**

**Supplementary Figure S7. Pairwise comparison of differentially abundant groups (DESeq2 analysis) between different lifestyles at a genus level**
